# Deep Learning–Assisted Diagnosis of Middle Ear Diseases in Otoendoscopy Across Clinician Experience Levels: Retrospective Reader Study

**DOI:** 10.2196/96134

**Published:** 2026-10-30

**Authors:** Se Eun Yi, Jihye Chae, Keunwoo Park, Yeon Joo Choi, Jihoon Kweon, Joong Ho Ahn

**Affiliations:** 1 Department of Otorhinolaryngology-Head and Neck Surgery, Asan Medical Center, University of Ulsan College of Medicine, 88 Olympic-ro 43-gil, Songpa-gu, Seoul, 05505, Republic of Korea, 82 2-3010-3717, 82 2-489-2773; 2 Department of Medical Science, Asan Medical Institute of Convergence Science and Technology, Asan Medical Center, University of Ulsan College of Medicine, Seoul, Republic of Korea; 3 Department of Biomedical Engineering, Asan Medical Center, University of Ulsan College of Medicine, Seoul, Republic of Korea

**Keywords:** deep learning, artificial intelligence, AI, middle ear disease, otoscopy, diagnostic accuracy, clinical decision support

## Abstract

**Background:**

Accurate diagnosis of middle ear diseases remains challenging due to the specialized nature of otoscopic interpretation and the global shortage of otolaryngologists.

**Objective:**

We aimed to evaluate the impact of a deep learning–based AI support system on the diagnostic performance of clinicians with varying levels of expertise, specifically focusing on its ability to bridge the gap between trainees and experts.

**Methods:**

In this retrospective reader study, a deep learning model (EfficientNet-B4) was developed using a large-scale dataset of 7335 endoscopic images. The model was designed for multi-class classification, simultaneously identifying primary categories (chronic otitis media, otitis media with effusion, or none [indicating the absence of the first two]) and secondary features (attic retraction, myringitis, otomycosis, and ventilation tube). To assess clinical impact, diagnostic performance was compared among 3 otology professors, 5 senior residents, and 5 junior residents using an independent clinical assessment set of 100 images. Participants performed diagnoses both with and without AI-generated predictions, with a time constraint of 1 minute per image to simulate real-world clinical pressure.

**Results:**

In 5-fold cross-validation, the deep learning model achieved 94.8% accuracy for the primary classification. The stand-alone AI achieved diagnostic accuracies ranging from 94.0% to 99.0% in the clinical assessment set. AI support significantly enhanced diagnostic accuracy across all groups for primary classification, with improvements of 11.0% for otology professors, 28.8% for senior residents, and 32.2% for junior residents (*P*<.001). Accuracy increased from 78.7% to 89.7% for otology professors, from 65.0% to 93.8% for senior residents, and from 54.4% to 86.6% for junior residents. The accuracy gap between the most experienced (otology professors) and least experienced (junior residents) groups was reduced from 24.3% to 3.1%. For secondary categories, significant improvements were observed in diagnosing attic retraction and myringitis among residents. AI-supported accuracy for attic retraction was higher in senior residents than that of the stand-alone AI, although the 95% CIs overlapped substantially, so the difference was not statistically significant.

**Conclusions:**

Our findings demonstrate that deep learning–based AI support substantially improves the diagnostic accuracy of middle ear diseases, particularly for less experienced clinicians. By narrowing the performance gap between trainees and experts, this system shows high potential as a valuable adjunct for clinical decision-making and medical education in otology.

## Introduction

Middle ear diseases, including chronic otitis media (COM) and otitis media with effusion (OME), are prevalent worldwide [1]. For example, OME is extremely common, with a prevalence of 80% to 90% in children, and early diagnosis is crucial due to the potential impact on speech and language development associated with hearing loss [2,3]. However, many countries face difficulties due to a shortage of otolaryngologists. In 64% of African countries, there is less than 1 otolaryngologist per million people [4]. Otoscopy, essential for diagnosing middle ear diseases [5], can present challenges even for specialists in achieving accurate interpretation. Due to the shortage of specialists, nonspecialists often diagnose middle ear diseases [4,6,7], which can lead to misdiagnoses and, consequently, inadequate treatment.

To address the challenge of diagnosing middle ear diseases, machine learning (ML) approaches for the diagnosis of middle ear diseases have been proposed for decades [8-10]. ML models can be used to accurately predict the presence or absence of middle ear effusions in pediatric patients and achieve significantly higher diagnostic accuracy than traditional otoscopy performed by experts [8]. A convolutional neural network was developed to automatically detect tympanic membrane (TM) and middle ear infections from endoscopic images, showing high accuracy and potential for clinical application [9]. The diagnostic performance of the ML models was comparable to that of experienced otolaryngologists and demonstrated that the imbalance problem due to disease prevalence could be mitigated through data augmentation [11]. Recent reviews and a meta-analysis have also reported the growing use of image-based AI for diagnosing middle ear diseases, with generally high diagnostic performance [12-14]. Recent studies have further evaluated the generalizability of these models and compared their performance with that of clinicians [15,16].

In a previous study [10], we developed a deep learning model capable of simultaneously diagnosing 2 or more diseases using a database of 6630 endoscopy images that included various diseases. Our deep learning model could predict 2 noncoexisting diseases (OME and COM) along with 4 concurrently detectable diseases (attic retraction, myringitis, otomycosis, and ventilation tube). The diagnostic accuracy ranged from 95.3% to 99.4%.

Although deep learning models have demonstrated high diagnostic performance, their accuracy can fluctuate based on training data and model architecture [11,17]. There is also a risk of misdiagnosis in cases with apparent characteristics. Consequently, there is growing interest in how ML models can enhance diagnostic performance by supporting clinical decisions. Recent studies have also examined human-AI collaboration in clinical diagnosis, including how AI recommendations influence physician decision-making and diagnostic performance [18,19]. In dermatology, for instance, decision support from deep learning systems improved diagnostic accuracy by 33% for both specialists and general practitioners [17]. However, in the field of endoscopic diagnosis for middle ear diseases, the impact of deep learning models on clinical decisions remains unexplored. This study makes 3 contributions. We quantify the effect of AI support on middle ear diagnosis across different levels of clinical experience; assess whether AI support narrows the diagnostic performance gap between trainees and experts; and characterize how clinicians respond to AI recommendations, including changes in diagnostic decisions after AI support.

## Methods

### Data Description

Endoscopic images were retrospectively collected at Asan Medical Center from January 2018 to December 2020 (Figure 1A). The final dataset comprised 7435 images, including 7335 images for model development and a separate clinical assessment set of 100 images. Each patient contributed 1 image to the dataset. Endoscopic examinations were performed for diagnostic purposes, and the captured images were stored in the hospital server without patient identification information.

Two experienced otologists independently and blindly annotated each collected image (Figure 1B). Only images with complete agreement between the 2 otologists were included in either the model development dataset or the clinical assessment set, and images with any discordant annotation were excluded. The excluded images were not retained, and their number was not recorded. No additional objective reference standard was used for the clinical assessment set. In the primary class, the collected images were annotated as one of the following: “COM,” “OME,” and “None” (indicating the absence of COM or OME). COM was defined as visible perforation of the TM. OME was defined as the presence of effusion in the middle ear cavity, which was characterized by the color change of the TM, such as amber, or the air fluid level in the middle ear cavity. In the secondary classes, the collected images were annotated as presence or absence of “attic retraction,” “myringitis,” “otomycosis,” and “ventilation tube.” Attic retraction was defined as the retracted TM or bony destruction in the attic. Myringitis was defined as any inflammation of the TM, including acute otitis media. Otomycosis was defined as the whitish debris or visible pores of fungus in the TM or external auditory canal. Ventilation tube was defined as a tube inserted through the TM. A myringotomy containing a ventilation tube was not considered a perforation in the annotation of COM. We expanded our database with more cases of attic retraction and otomycosis, which had shown relatively lower diagnostic performance in our previous study [10]. The distribution of cases across the primary and secondary classes is summarized in Figure 1B.

The clinical assessment set did not overlap with the model development dataset. None of the 100 images in the clinical assessment set were included in the training, validation, or test folds used to develop the 5 models comprising the ensemble. For this set, cases with a primary class of “None” and negative findings for all secondary classes were excluded to avoid overrepresentation of normal cases. The number of cases for each disease category was determined based on the class distribution used in our previous study [10], and images were randomly selected within these categories. No images were excluded based on image quality assessment for either model training or clinician evaluation. The clinical assessment set size was determined pragmatically by considering reader burden and the need to evaluate multiple clinician groups both before and after AI support.

**Figure 1.**
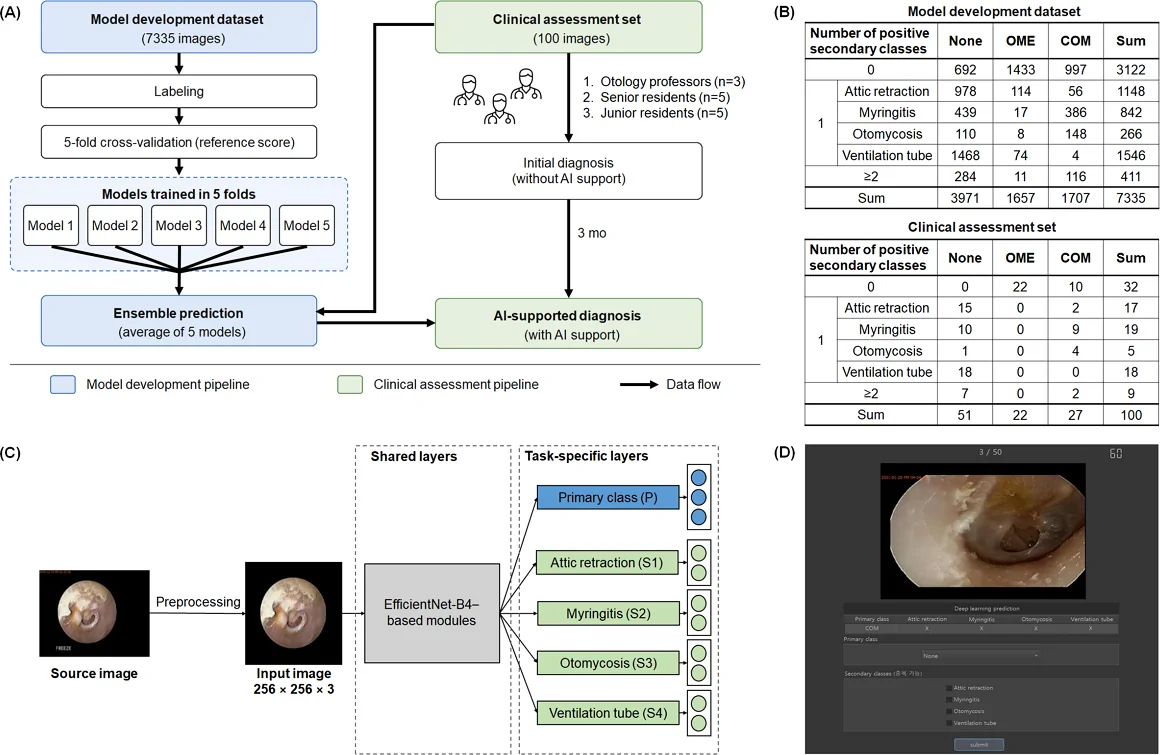
(A) Schematic diagram for comparison of initial and AI-supported diagnoses, (B) distribution of otoendoscopic images across primary and secondary classes in the model development dataset (top) and the clinical assessment set (bottom), (C) deep learning network for multi-class classification, and (D) user interface for clinical assessment. “None” indicates the absence of chronic otitis media (COM) and otitis media with effusion (OME).

### Construction of AI Support Using Deep Learning

The deep learning network architecture in this study was based on our previous work [10]. EfficientNet-B4 [20] was customized to incorporate both shared and task-specific layers, enabling precise predictions of concurrently existing diseases in the TM (Figure 1C). The deep learning model classified an endoscopic image into one of the primary classes: OME, COM, and “None.” Simultaneously, it determined the presence or absence of 4 secondary classes: attic retraction, myringitis, otomycosis, and ventilation tube (Figure 1).

RGB images reformatted to 256 × 256 × 3 with circular cropping were fed to the deep networks as inputs. Circular cropping removed only the noninformative dark periphery outside the otoendoscopic field of view while retaining the TM and the surrounding external auditory canal. The pretrained weights from ImageNet were applied for transfer learning. Categorical cross-entropy loss was used for the mutually exclusive primary classes, whereas binary cross-entropy loss was used for each of the 4 secondary classes because the secondary classes were not mutually exclusive. To prevent overfitting, data augmentation methods were used: random rotation (−90° to 90°), shift (0%‐20% of the image size in the horizontal and vertical axes), magnification (0%‐20%), horizontal flip, brightness modification (0%‐20%), and resolution reduction (0%‐50%). The models were trained for 200 epochs with a differential learning rate, which was initially set as 10^–3^ and decreased by half when the validation loss stagnated for 50 epochs. To assess the model performance more objectively and robustly, 5-fold cross-validation was performed using 7335 images (Figure 1A). Five-fold cross-validation was performed with each patient’s single image assigned to only 1 fold, thereby preventing patient overlap across folds. For 5-fold cross-validation, 3 folds were used for training, 1 fold was used for validation, and 1 fold was used for testing. The validation and test folds were permutatively changed across the 5 folds. The reported cross-validation performance metrics were calculated using the test set, not the training or validation data.

The evaluation metrics for each label were precision, sensitivity (recall), specificity, and Dice similarity coefficient (DSC), which were defined as precision = true positives [TPs]/(TPs + false positives [FPs]), sensitivity = TPs/(TPs + false negatives [FNs]), specificity = true negatives [TNs]/(FPs + TNs), and DSC = 2 × precision × recall/(precision + recall). The per-class accuracy was calculated by dividing the sum of TPs and TNs by the total number of images in a fold.

### Impact of AI Support on Diagnostic Decision

To analyze the impact of deep learning predictions on diagnostic decisions, we compared diagnoses made without AI support (“initial diagnosis”) to those made after referring to deep learning predictions (“AI-supported diagnosis”). The AI-supported diagnosis was conducted 3 months after the initial diagnosis. The order of the 100 images was randomized during the second reading session to reduce recall bias. For the AI-supported diagnosis, clinicians were provided with predictions generated using a soft-voting ensemble of 5 deep learning models trained during the cross-validation. The predicted probabilities were averaged across the 5 models to generate the final AI predictions. During the AI-supported assessment, clinicians reviewed the AI predictions before making their final diagnostic decisions. The AI predictions were presented as categorical outputs for the primary and secondary classes. No confidence scores or probabilities, heat maps, saliency maps, or other explanatory visualizations were provided to the clinicians. We compared the diagnostic performance across 3 groups: otology professors (n=3), senior residents (n=5), and junior residents (n=5). For each clinician group, sensitivity, specificity, positive predictive value (PPV), and negative predictive value (NPV) were additionally calculated for the primary and secondary classes. Diagnoses for the clinical assessment set were made using still images identical to the input of deep learning model, with participants given 1 minute per image.

To characterize clinician responses to AI recommendations, we additionally analyzed cases in which the initial diagnosis differed from the AI prediction for the primary class. An AI recommendation was considered accepted when the AI-supported diagnosis matched the AI prediction and rejected when the AI-supported diagnosis did not match the AI prediction. We also categorized each diagnosis according to the change in correctness from the initial to the AI-supported assessment as incorrect to correct, correct to incorrect, correct to correct, or incorrect to incorrect.

### Statistical Analysis

All statistical analyses were performed using SAS software (version 9.4; SAS Institute). Assessment times for the initial and AI-supported diagnoses were summarized using the median and IQR. The 95% CIs for accuracy, DSC, sensitivity, specificity, PPV, and NPV were estimated using the percentile bootstrap method with 2000 replicates. For each replicate, images were sampled with replacement to generate a bootstrap sample of the same size as the original dataset, and the performance metrics were recalculated. Because the resampling unit was the image, these CIs do not account for between-clinician variability. *P* values for accuracy were calculated using generalized estimating equations with image as the clustering unit to model repeated diagnostic assessments of the same image across clinicians and reading sessions. “Clinician” was not included as a clustering factor. *P*<.05 was considered statistically significant.

### Ethical Considerations

This study was approved by the institutional review board of Asan Medical Center (2021‐0837), and the requirement for informed consent for the retrospective use of otoendoscopic images was waived. All study images were deidentified before analysis, and the investigators had no access to personal identifying information. Written informed consent was obtained from all clinician readers before participation. No compensation was provided to the clinician readers. No images included in the manuscript contain identifiable facial features or personal information. The study was conducted in accordance with the Declaration of Helsinki.

## Results

### Diagnostic Performance of Deep Learning Models in Cross-Validation

Table 1 shows the diagnostic performance of deep learning models in the 5-fold cross-validation using 7335 images. In predicting the primary class, both accuracy and DSC were 94.8%. Among the secondary classes, ventilation tube was the most accurately diagnosed (99.5%), followed by otomycosis and attic retraction. Myringitis had a relatively lower predictive accuracy of 95.0%.

**Table 1.** Diagnostic performance of deep learning models in 5-fold cross-validation (“reference score” in Figure 1A).

Class	Accuracy (%; 95% CI)	DSC^a^ (%; 95% CI)	Sensitivity (%; 95% CI)	Specificity (%; 95% CI)
Primary class	94.8 (94.3‐95.3)	94.8 (94.3‐95.3)	94.8 (94.3‐95.3)	95.6 (95.0‐96.0)
Attic retraction	97.0 (96.6‐97.3)	91.4 (90.3‐92.5)	88.6 (87.0‐90.3)	98.8 (98.5‐99.1)
Myringitis	95.0 (94.5‐95.5)	84.1 (82.4‐85.7)	83.7 (81.4‐85.8)	97.1 (96.7‐97.6)
Otomycosis	98.7 (98.4‐98.9)	88.5 (86.1‐90.7)	81.9 (78.3‐85.4)	99.8 (99.7‐99.9)
Ventilation tube	99.5 (99.3‐99.6)	98.8 (98.4‐99.2)	98.5 (97.9‐99.1)	99.7 (99.6‐99.9)

a DSC: Dice similarity coefficient.

### Diagnostic Performance of AI Support for the Clinical Assessment Set

AI support suggested diagnoses with accuracy ranging from 94.0% (myringitis) to 99.0% (ventilation tube), as shown in Table 2. When compared to cross-validation, the accuracies for individual disease identification varied by 0.2% to 1.7%.

**Table 2.** Comparison of diagnostic accuracy between initial and AI-supported diagnoses^a^.

Class	AI support score (%; 95% CI)	Otology professors (n=3)			Senior residents (n=5)			Junior residents (n=5)		
		Initial diagnoses (%; 95% CI)	AI-supported diagnoses (%; 95% CI)	*P* value	Initial diagnoses (%; 95% CI)	AI-supported diagnoses (%; 95% CI)	*P* value	Initial diagnoses (%; 95% CI)	AI-supported diagnoses (%; 95% CI)	*P* value
Primary class	95.0 (90.0‐99.0)	78.7 (74.0‐83.0)	89.7 (85.3‐93.3)	<.001	65.0 (58.2‐71.8)	93.8 (91.0‐96.4)	<.001	54.4 (46.6‐62.0)	86.6 (82.6‐90.2)	<.001
Attic retraction	98.0 (95.0‐100.0)	96.3 (92.7‐99.0)	97.3 (94.3‐99.3)	.25	96.6 (94.2‐98.6)	99.0 (97.8‐99.8)	<.001	94.2 (90.8‐97.0)	98.4 (97.0‐99.4)	<.001
Myringitis	94.0 (89.0‐98.0)	87.0 (81.3‐92.0)	91.7 (87.7‐95.3)	.01	87.6 (82.2‐92.4)	91.4 (87.0‐95.2)	.03	75.4 (68.0‐82.6)	88.4 (83.0‐93.2)	<.001
Otomycosis	97.0 (93.0‐100.0)	96.3 (92.7‐99.0)	96.3 (93.7‐98.7)	>.99	96.2 (93.0‐98.6)	98.2 (96.0‐99.8)	.08	96.0 (93.0‐98.4)	95.8 (92.8‐98.4)	.85
Ventilation tube	99.0 (97.0‐100.0)	99.3 (98.3‐100.0)	99.7 (99.0‐100.0)	.57	99.8 (99.4‐100.0)	99.6 (99.0‐100.0)	.57	99.2 (98.4‐99.8)	98.4 (97.0‐99.4)	.19

a *P* values represent comparisons between the initial and AI-supported diagnoses within each clinician group.

### Initial Diagnosis of Clinicians for the Clinical Assessment Set

The diagnostic accuracy for the primary class demonstrated a positive correlation with clinical experience (*P*<.001 for otology professors vs senior residents, otology professors vs junior residents, and senior vs junior residents; Table 3). The junior resident group showed a significantly lower accuracy of 54.4%. For all secondary classes, no significant differences were found between the otology professor and senior resident groups. On the other hand, in the diagnosis of myringitis, the junior resident group showed an accuracy difference of more than 11.6% compared to both the otology professor and senior resident groups (*P*<.001).

**Table 3.** Comparison of diagnostic accuracy between groups with varying levels of expertise^a^.

	Diagnostic accuracy (%; 95% CI)			*P* value		
	OPs^b^ (n=3)	SRs^c^ (n=5)	JRs^d^ (n=5)	OPs vs SRs	OPs vs JRs	SRs vs JRs
Initial diagnosis						
Primary class	78.7 (74.0‐83.0)	65.0 (58.2‐71.8)	54.4 (46.6‐62.0)	<.001	<.001	<.001
Attic retraction	96.3 (92.7‐99.0)	96.6 (94.2‐98.6)	94.2 (90.8‐97.0)	.83	.20	.002
Myringitis	87.0 (81.3‐92.0)	87.6 (82.2‐92.4)	75.4 (68.0‐82.6)	.73	<.001	<.001
Otomycosis	96.3 (92.7‐99.0)	96.2 (93.0‐98.6)	96.0 (93.0‐98.4)	.85	.83	.89
Ventilation tube	99.3 (98.3‐100.0)	99.8 (99.4‐100.0)	99.2 (98.4‐99.8)	.33	.83	.22
AI-supported diagnosis						
Primary class	89.7 (85.3‐93.3)	93.8 (91.0‐96.4)	86.6 (82.6‐90.2)	.03	.17	<.001
Attic retraction	97.3 (94.3‐99.3)	99.0 (97.8‐99.8)	98.4 (97.0‐99.4)	<.001	.20	.28
Myringitis	91.7 (87.7‐95.3)	91.4 (87.0‐95.2)	88.4 (83.0‐93.2)	.87	.03	.04
Otomycosis	96.3 (93.7‐98.7)	98.2 (96.0‐99.8)	95.8 (92.8‐98.4)	.12	.66	.051
Ventilation tube	99.7 (99.0‐100.0)	99.6 (99.0‐100.0)	98.4 (97.0‐99.4)	.88	.14	.09

a *P* values were calculated using generalized estimating equations for pairwise comparisons between clinician groups.

b OP: otology professor.

c SR: senior resident.

d JR: junior resident.

### AI-Supported Diagnosis of Clinicians for the Clinical Assessment Set

AI support significantly improved the diagnostic accuracy of the primary class by 11.0%, 28.8%, and 32.2% for the otology professor, senior resident, and junior resident groups, respectively (*P*<.001; Table 2). The senior resident group achieved the highest accuracy (*P*=.03 for otology professors vs senior residents; Table 3), whereas the difference between the otology professor and junior resident groups was reduced to 3.1% (*P*=.17; Table 3). Among the secondary classes, both the senior and junior resident groups demonstrated significant performance improvements in diagnosing attic retraction and myringitis (Table 2). For attic retraction, AI-supported accuracy was higher in the senior resident group than that of stand-alone AI (99.0%, 95% CI 97.8%‐99.8% vs 98.0%, 95% CI 95.0%‐100%), although the 95% CIs overlapped substantially. Regarding otomycosis and ventilation tube diagnoses, accuracy differences among the 3 groups ranged from –0.8% to 2%, but these changes were not statistically significant. Sensitivity, specificity, PPV, and NPV for each clinician group can be found in Multimedia Appendix 1. The median assessment time was 3 seconds in both the initial (IQR 2‐5 seconds) and AI-supported (IQR 2‐6 seconds) sessions.

### Clinician Responses to AI Recommendations

Among 1300 clinician image assessments for the primary class, the initial diagnosis differed from the AI recommendation in 499 cases (Table 4). Clinicians accepted the AI recommendation in 79.2% (395/499) of cases and rejected it in 20.8% (104/499) of cases. The acceptance rate was 65.8% (50/76) for otology professors, 84.0% (157/187) for senior residents, and 79.7% (188/236) for junior residents. Final diagnoses were correct in 95.9% (379/395) of accepted cases and 31.7% (33/104) of rejected cases. Among rejected recommendations, clinicians retained their initial diagnosis in 90 cases and selected a diagnosis different from both the initial diagnosis and the AI recommendation in 14 cases. Across all 1300 assessments, 384 initial errors were corrected after AI support, whereas 46 initially correct diagnoses became incorrect. Of these 46 cases, 13 occurred when the initial diagnosis differed from the AI recommendation and 33 occurred when it matched the AI prediction.

Acceptance of AI recommendations differed across experience groups. When the initial diagnosis differed from the AI prediction, otology professors accepted the AI recommendation in 65.8% (50/76) of cases compared with 84.0% (157/187) for senior residents and 79.7% (188/236) for junior residents. When clinicians rejected the AI recommendation, the final diagnosis was correct in 42.3% (11/26) of cases for otology professors and 46.7% (14/30) for senior residents, whereas it was correct in only 16.7% (8/48) of cases for junior residents.

**Table 4.** Clinician responses to AI recommendations for the primary class.

Clinician response	OPs^a^	SRs^b^	JRs^c^	Overall
Total assessments, n	300	500	500	1300
Initial diagnosis differed from AI recommendation, n	76	187	236	499
AI recommendation accepted, n/N (%)	50/76 (65.8)	157/187 (84.0)	188/236 (79.7)	395/499 (79.2)
AI recommendation rejected, n/N (%)	26/76 (34.2)	30/187 (16.0)	48/236 (20.3)	104/499 (20.8)
Correct after acceptance, n/N (%)	48/50 (96)	152/157 (96.8)	179/188 (95.2)	379/395 (95.9)
Correct after rejection, n/N (%)	11/26 (42.3)	14/30 (46.7)	8/48 (16.7)	33/104 (31.7)
Incorrect→correct, n/N (%)	48/300 (16.0)	155/500 (31)	181/500 (36.2)	384/1300 (29.5)
Correct→incorrect, n/N (%)	15/300 (5)	11/500 (2.2)	20/500 (4)	46/1300 (3.5)
Correct→correct, n/N (%)	221/300 (73.7)	314/500 (62.8)	252/500 (50.4)	787/1300 (60.5)
Incorrect→incorrect, n/N (%)	16/300 (5.3)	20/500 (4)	47/500 (9.4)	83/1300 (6.4)

a OP: otology professor.

b SR: senior resident.

c JR: junior resident.

## Discussion

### Major Findings

In this study, we developed AI support for the diagnosis of middle ear diseases using a deep learning model trained on a large-scale database of 7335 endoscopic images. The data imbalance issue arising from different prevalence rates was mitigated by using our expanded database. The trained models effectively reduced the disparity in diagnostic performance across individual diseases, consequently enhancing the quality of our AI support. For a separate clinical assessment set of 100 images, AI support improved diagnostic performance across 3 groups—otology professors, senior residents, and junior residents—while also reducing the disparity in diagnostic accuracy due to different levels of clinical expertise. In the diagnosis of attic retraction, referring to AI predictions enabled senior residents to achieve higher accuracy than that of stand-alone AI, although the 95% CIs overlapped substantially.

### Clinical Impact of AI Support

The diagnostic accuracy of clinicians in this study ranged from 54.4% to 78.7% for the primary class, which is comparable to the performance reported in our previous study [10]. By referring to the AI support, the diagnostic accuracy of clinicians improved to between 86.6% and 93.8%. Recent studies have also shown that AI can outperform clinicians in middle ear disease diagnosis [16]. AI assistance has also been shown to reduce workload in medical image interpretation [21]. The junior resident group, which had the lowest diagnostic accuracy in the initial diagnosis, showed the highest propensity to alter decisions after referring to AI support (Figure 2). The additional analysis showed that improvement after AI support was primarily associated with correction of initially incorrect diagnoses, although some initially correct diagnoses became incorrect after AI support. The 33 correct-to-incorrect changes that occurred when the initial diagnosis matched the AI prediction likely reflect intrareader variability over the interval rather than an effect of AI support. Clinician responses to AI recommendations also differed according to clinical experience. Senior and junior residents were more likely than otology professors to accept AI recommendations, whereas junior residents were less likely to make a correct final diagnosis when they rejected the AI recommendation. These findings suggest that the role of AI support may differ according to the clinician’s level of experience. Considering the characteristics of middle ear diagnosis, where multiple diseases are concurrently detectable, this finding demonstrates the potential of AI support in augmenting clinical decision-making, particularly in cases that pose diagnostic challenges for less experienced clinicians, including nonotologists.

**Figure 2.**
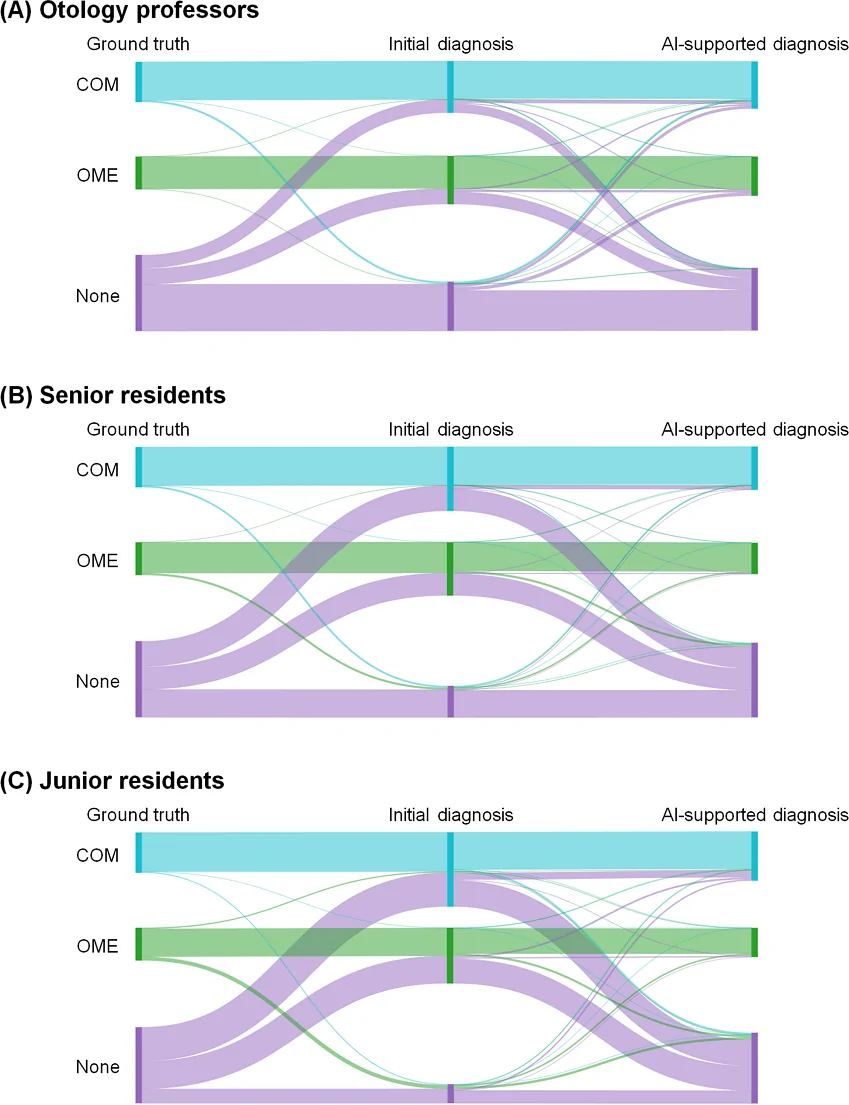
Changes in diagnostic performance of the primary class through referencing AI support. “None” indicates the absence of chronic otitis media (COM) and otitis media with effusion (OME).

### Use of AI Support in Medical Education

The deep learning models and evaluation methods used in this study can also be applied to educational simulations. Previous research has demonstrated that otoscope simulation enhances confidence in diagnosing otological diseases compared to conventional training methods [5]. With the evaluation system used in this study, trainees can be exposed to a diverse array of endoscopic images in a short period. Furthermore, the integration of deep learning predictions in constructing simulation data offers a novel approach to medical education in otology, potentially accelerating the learning curve and improving diagnostic skills across various levels of expertise.

### Diagnostic Errors With AI Support

Diagnostic errors in the primary class predominantly manifested as misclassifications of “None” (absence of COM or OME) as disease positive, whereas the opposite scenario was exceedingly rare (Figure 2). This pattern likely stemmed from the exclusion of cases in the clinical assessment set where the primary class was “None” and all secondary classes were negative, which represented cases without COM, OME, or any secondary finding (Figure 1B). Intriguingly, within the otology professor group, there were instances in which images initially correctly diagnosed as “None” were reclassified as COM or OME after referring to AI support. This suggested the possibility that diagnostic capabilities may be blurred by AI support. Consequently, the otology professor group exhibited a higher rate of FPs for OME than the senior and junior resident groups. This finding is consistent with growing concerns that AI support may improve diagnostic performance while also creating risks of automation bias [22]. In our study, the increased false-positive diagnoses of OME among otology professors after AI support suggest that even experienced clinicians may be influenced by AI suggestions in selected cases. This finding also suggests that AI support should not be assumed to provide uniform benefits across levels of clinical expertise. Therefore, clinicians should interpret AI outputs in the context of their own assessment and consider them as supportive information.

AI assistance also carries potential risks. Beyond the automation bias discussed above, reliance on AI may lead clinicians to defer to the model even when their independent judgment would be correct. Habitual use of diagnostic AI during training may also affect the development of independent diagnostic skills. These effects could not be evaluated with the current study design and warrant prospective and longitudinal investigation.

### Limitations

First, our deep learning models for AI support were trained on a database from a single medical center, potentially limiting their generalizability. Because the dataset was fully anonymized before analysis, demographic information such as age and sex was unavailable, and the demographic representativeness of the dataset could not be evaluated. In addition, exclusion of images with discordant annotations may have removed diagnostically challenging cases and led to overestimation of both model and clinician performance. Future studies should incorporate multicenter data encompassing diverse patient demographics and a variety of imaging devices.

Second, the coverage of AI support needs to expand to include additional middle ear diseases such as congenital cholesteatoma and tympanosclerosis plaque. Furthermore, our results indicated relatively lower diagnostic accuracy for myringitis and otomycosis. The input images were resized to 256 × 256 × 3 rather than using the original high-resolution images to support efficient model training and potential real-time application. Although the model showed high overall classification performance, this downsampling may have reduced fine-grained visual details used for diagnosis. These limitations may reduce the applicability of the system across a broader range of middle ear diseases. EfficientNet-B4 also operates as a black-box model, and the image features underlying its predictions are not directly interpretable.

Third, the clinical assessment set was disease enriched because cases with a primary class of “None” and negative findings for all secondary classes were excluded. Therefore, it did not reflect the prevalence of normal ears in routine clinical practice, which may have affected performance estimates and contributed to the observed false-positive rate. The clinical assessment was based on 100 images. A set of this size limits the precision with which the diagnostic improvements are estimated and increases sampling variability, particularly for secondary categories with few positive cases. Although AI support improved primary-class accuracy across all 3 experience groups, the magnitude of the observed improvements should be interpreted with caution and confirmed in a larger assessment set. The clinical assessment involved a small and unbalanced clinician sample (3 otology professors, 5 senior residents, and 5 junior residents). These small group sizes, particularly for the otology professor group, limit the statistical reliability of the between-group comparisons. Because the bootstrap resampled images only, the resulting CIs do not reflect between-clinician variability. The generalized estimating equations models accounted for repeated assessments of the same image. Correlation among readings by the same clinician was not modeled. Therefore, uncertainty in the between-group comparisons may be underestimated, particularly given the small and unbalanced reader sample.

Fourth, the clinical assessment used single static images, which may not fully reflect real-world otoendoscopic evaluation based on dynamic examination and clinical context. The AI support was evaluated using selected static otoendoscopic images, which may introduce frame selection bias. In real practice, diagnostic performance may also depend on how well the clinician captures the image, which could influence AI-assisted diagnosis. Image quality in real-time use may also vary because of differences in illumination, focus, artifacts, and anatomical variations. The robustness of the model under conditions not represented in the study data was not specifically evaluated. The 1-minute time limit was reached in only 3 AI-supported readings and was therefore unlikely to have materially affected the comparison between the 2 sessions.

Fifth, all clinicians performed the initial diagnosis first and the AI-supported diagnosis 3 months later. This fixed order means that the AI support effect cannot be fully separated from a learning effect over the interval or a residual memory effect for the same images. This interval may also explain the correct-to-incorrect changes that occurred when the initial diagnosis matched the AI prediction. The magnitude of the observed improvement, particularly in the resident groups, should therefore be interpreted with caution. Future studies should counterbalance the reading order across clinicians to better isolate the effect of AI support.

Sixth, this study assessed changes in diagnostic decisions before and after exposure to AI predictions but did not directly evaluate how clinicians interpreted or weighted the AI recommendations. Therefore, the specific human-AI decision-making processes underlying these changes remain unclear, including whether providing confidence or uncertainty information would alter clinicians’ decision patterns, particularly for borderline cases.

Seventh, AI support’s utility for nonotologists remains unexplored. This limits the generalizability of our findings beyond otologists. Given that primary care physicians often encounter middle ear diseases, validating the AI support’s effectiveness in these broader clinical contexts would be valuable.

### Conclusions

We developed an AI support system for middle ear disease diagnosis using an expanded database of 7335 endoscopic images, demonstrating potential to enhance diagnostic performance across different levels of clinical expertise. With AI support, the primary class accuracy gap between otology professors and junior residents narrowed from 24.3% to 3.1%, and junior residents’ sensitivity increased from 66.8% to 87.2% for the primary class and from 21.4% to 60.0% for myringitis. The diagnostic improvements were most evident among junior residents, who showed the highest tendency to modify their initial diagnoses based on AI support, reaching a level of diagnostic performance comparable to that of otology professors. These results were obtained at a single center with a specific endoscope type and patient population. Although AI support narrowed the trainee-expert performance gap in this setting, its generalizability to the lower-resource environments in which such support is most needed remains to be established, and multicenter validation across diverse hardware and patient populations is required before broad policy implications are drawn.

## Supplementary material

10.2196/96134 Multimedia Appendix 1 Sensitivity, specificity, positive predictive value, and negative predictive value of clinician diagnoses before and after AI support.

## Abbreviations

COM: chronic otitis media
DSC: Dice similarity coefficient
FN: false negative
FP: false positive
ML: machine learning
NPV: negative predictive value
OME: otitis media with effusion
PPV: positive predictive value
TM: tympanic membrane
TN: true negative
TP: true positive
