# Supplementary material for: Deep Learning–Assisted Diagnosis of Middle Ear Diseases in Otoendoscopy Across Clinician Experience Levels: Retrospective Reader Study
Source: JMIR Med Inform. 2026 Oct 30;14:e96134. doi: 10.2196/96134 (PMC13722516; doi:10.2196/96134)
Supplement: Supplementary file 1 [file medinform-v14-e96134-s001.docx]

**Table S1**. Sensitivity, specificity, positive predictive value, and negative predictive value of clinician diagnoses before and after AI support.

|  | **Sensitivity** | | | **Specificity** | | | **PPV** | | | | **NPV** | | | |
| --- | --- | --- | --- | --- | --- | --- | --- | --- | --- | --- | --- | --- | --- | --- |
|  | **OP (n=3)** | **SR (n=5)** | **JR (n=5)** | **OP (n=3)** | **SR (n=5)** | **JR (n=5)** | **OP (n=3)** | | **SR (n=5)** | **JR (n=5)** | **OP (n=3)** | **SR (n=5)** | **JR (n=5)** |  |
| **Initial** | | | | | | | | | | | | | | |
| Primary class | 85.0  (82.4–87.5) | 75.2  (72.2–78.0) | 66.8  (63.2–70.5) | 90.2  (88.3–92.1) | 83.9  (81.4–86.4) | 79.0  (76.2–81.9) | | 78.7  (72.3–83.9) | 68.4  (61.8–74.0) | 59.1  (52.7–65.8) | 89.4  (86.3–92.1) | 84.9  (81.5–87.9) | 81.8  (78.6–85.0) |  |
| Attic retraction | 84.3  (66.7–98.0) | 83.5  (72.9–93.3) | 74.1  (57.6–87.1) | 98.8  (97.4–100.0) | 99.3  (98.4–100.0) | 98.3  (96.6–99.7) | | 93.5  (84.1–100.0) | 95.9  (89.4–100.0) | 90.0  (77.5–97.9) | 96.9  (93.1–99.6) | 96.7  (93.8–98.9) | 94.9  (91.0–98.0) |  |
| Myringitis | 53.6  (40.4–66.7) | 61.4  (47.5–74.0) | 21.4  (14.4–29.1) | 100.0  (100.0–100.0) | 97.8  (95.6–99.4) | 96.4  (93.6–98.6) | | 100.0  (100.0–100.0) | 91.5  (82.0–97.9) | 69.8  (50.0–87.5) | 84.7  (77.6–90.9) | 86.7  (80.0–92.6) | 75.9  (67.7–84.0) |  |
| Otomycosis | 63.0  (37.0–86.7) | 66.7  (42.9–87.5) | 71.1  (47.5–92.5) | 99.6  (98.8–100.0) | 99.1  (97.9–100.0) | 98.5  (97.1–99.6) | | 94.4  (76.9–100.0) | 88.2  (68.0–100.0) | 82.1  (58.8–94.8) | 96.5  (92.7–99.3) | 96.8  (93.7–99.2) | 97.2  (94.3–99.6) |  |
| Ventilation tube | 98.6  (95.2–100.0) | 99.1  (97.0–100.0) | 97.4  (94.2–100.0) | 99.6  (98.6–100.0) | 100.0  (100.0–100.0) | 99.7  (99.2–100.0) | | 98.6  (94.7–100.0) | 100.0  (100.0–100.0) | 99.1  (96.8–100.0) | 99.6  (98.6–100.0) | 99.7  (99.2–100.0) | 99.2  (98.2–100.0) |  |
| **AI-supported** | | | | | | | | | | | | | | |
| Primary class | 92.9  (90.4–95.4) | 93.9  (90.8–96.7) | 87.2  (82.7–90.9) | 95.3  (93.4–97.0) | 96.6  (95.0–98.0) | 92.9  (90.7–94.9) | | 88.4  (83.0–92.8) | 93.8  (90.3–96.4) | 86.9  (82.3–90.7) | 94.1  (91.5–96.3) | 96.5  (94.6–97.9) | 92.5  (90.0–94.8) |  |
| Attic retraction | 86.3  (70.8–98.2) | 95.3  (89.2–100.0) | 92.9  (86.7–98.3) | 99.6  (98.8–100.0) | 99.8  (99.2–100.0) | 99.5  (98.8–100.0) | | 97.8  (92.0–100.0) | 98.8  (95.2–100.0) | 97.5  (92.9–100.0) | 97.3  (94.0–99.6) | 99.0  (97.5–100.0) | 98.6  (97.0–99.8) |  |
| Myringitis | 73.8  (61.3–85.3) | 72.9  (60.8–83.5) | 60.0  (47.1–73.1) | 98.6  (96.9–100.0) | 98.6  (96.4–100.0) | 99.4  (98.6–100.0) | | 95.4  (88.7–100.0) | 95.3  (87.5–100.0) | 97.7  (93.7–100.0) | 90.6  (84.9–95.5) | 90.3  (85.0–95.2) | 86.5  (79.7–92.3) |  |
| Otomycosis | 81.5  (60.0–100.0) | 82.2  (60.0–100.0) | 73.3  (55.0–91.4) | 97.8  (96.0–99.3) | 99.8  (99.3–100.0) | 98.0  (95.6–99.6) | | 78.6  (53.3–93.5) | 97.4  (88.1–100.0) | 78.6  (52.2–96.0) | 98.2  (95.6–100.0) | 98.3  (95.8–100.0) | 97.4  (94.6–99.4) |  |
| Ventilation tube | 100.0  (100.0–100.0) | 98.3  (95.6–100.0) | 95.7  (91.8–99.0) | 99.6  (98.5–100.0) | 100.0  (100.0–100.0) | 99.2  (97.9–100.0) | | 98.6  (94.7–100.0) | 100.0  (100.0–100.0) | 97.3  (92.1–100.0) | 100.0  (100.0–100.0) | 99.5  (98.6–100.0) | 98.7  (97.4–99.7) |  |

CI, confidence interval; PPV, positive predictive value; NPV, negative predictive value; OP, otology professor; SR, senior resident; JR, junior resident. For the primary class, sensitivity, specificity, PPV, and NPV were macro-averaged across COM, OME, and None. Values are presented as % (95% CI).
